# Sulfur metabolism and immune-microbial networks across oral niches in periodontal disease

**DOI:** 10.1128/spectrum.03008-25

**Published:** 2026-08-31

**Authors:** A. S. Stephen, V. Nagala, B. Fattah, N. Dhadwal, C. Gonzales-Marin, D. G. Gillam, D. J. Bradshaw, G. R. Burnett, R. P. Allaker

**Affiliations:** 1 Institute of Dentistry, Queen Mary University of London 65976, London, United Kingdom; 2 Haleon 622496 , Weybridge, Surrey, United Kingdom; Universidad de Sevilla, Seville, Spain

**Keywords:** hydrogen sulfide, immune-metabolic networks, cytokines, oral microbiome, periodontitis, gingivitis

## Abstract

Volatile sulfur compounds (VSCs) integrate microbial metabolism with local inflammation in periodontal disease. We profiled five oral niches (saliva, tongue, subgingival, supragingival, and interdental plaque) across clinical health, gingivitis, and periodontitis by combining direct VSC measurements (subgingival and oral headspace H_2_S/CH_3_SH), functional cysteine/methionine degradation assays, 16S rRNA profiling, gingival crevicular fluid cytokines, and targeted qPCR. Subgingival H_2_S concentrations were significantly elevated in periodontitis after adjusting for age, sex, plaque index, and subgingival bacterial load (β = 1.24, *P* = 0.03), and tracked shifts in community composition and the cytokine milieu. Oral headspace CH_3_SH increased with disease, whereas headspace H_2_S showed a bimodal pattern (health and periodontitis). In cysteine assays, subgingival and tongue biofilms were the most efficient H_2_S producers per mg protein; this ranking was preserved after normalization to total bacteria by qPCR. Metagenome predictions indicated enhanced sulfur metabolism in disease, particularly in subgingival plaque, with relative enrichment of SAM-cycle/methionine biosynthesis pathways in health. Methionine degradation to CH_3_SH increased with disease severity, shifting from subgingival sites in health to interdental/supragingival plaque in disease, and occurring most frequently in saliva. Correlation networks revealed niche- and diagnosis-specific coupling among VSCs, cytokines, and taxa, including associations of *Capnocytophaga*, *Fusobacterium*, *Prevotella*, and *Corynebacterium*, with IL-1β, IL-4, IL-8, and MCP-1. Together, these data identify the subgingival crevice as a disproportionate source of sulfide and show that sulfur metabolism is spatially organized and disease-responsive. We show that subgingival H_2_S as a functional marker that integrates microbial dysbiosis and inflammation.

**IMPORTANCE:**

We asked how metabolism, microbes, and immunity fit together during gum disease. Using a systems approach across five oral sites, we combined sulfur metabolite measurements, functional assays, microbiome profiling, and cytokine data, and analyzed them as one network. The result is a comprehensive map showing that sulfur metabolism is spatially organized, disease-responsive, and tightly coupled to local immune signals, with the subgingival niche playing an outsized role. Further, this integrated readout turns sulfur metabolism into a useful window on dysbiosis and inflammation and offers a path toward simple monitoring of periodontal disease, such as point-of-care sensors detecting subgingival hydrogen sulfide or methanethiol, or functional assays in which a methionine rinse is followed by measurement of oral headspace gases to assess microbial sulfur metabolism.

## INTRODUCTION

Volatile sulfur compounds (VSCs), including hydrogen sulfide (H_2_S) and methanethiol (CH_3_SH), are metabolic end products of bacterial amino acid degradation and are frequently associated with oral malodor (1–3). VSCs also offer a metabolic window into the ecological state of the oral microbiome. Their production depends on the prevalence, spatial distribution, and activity of specific anaerobes that harbor sulfur-metabolic enzymes such as the cysteine desulfhydrases and methionine gamma lyases (4–6). Early work linked VSC generation chiefly to the tongue dorsum because of its extensive surface area and microbial biomass (7). Studies have measured appreciable sulfide inside periodontal pockets and showed that subgingival VSC levels rise with disease severity (8–10). A higher CH_3_SH:H_2_S ratio in periodontitis suggests enrichment of methionine-degrading taxa during dysbiosis (11–13).

Genomic surveys reveal that only a subset of oral taxa possess the genes required for methionine or cysteine degradation (8, 14). Many of these taxa are members of the “orange” and “red” periodontal complexes and expand in gingivitis and periodontitis at the expense of health-associated commensals (15, 16). Understanding sulfide flux in the subgingival oral niches is significant for several reasons. First, VSCs have cytotoxic potential and can modulate host tissue responses, potentially exacerbating tissue inflammation (4, 17). Second, their production reflects the presence and activity of specific microbial networks that thrive under dysbiotic conditions. Third, local VSC concentrations may serve as functional biomarkers linking microbial metabolism to inflammatory status. Despite this potential, few studies have directly quantified subgingival VSCs alongside comprehensive microbiome and immune profiling.

Cysteine and methionine degradation assays can measure the latent capacity of microbial communities to generate sulfur metabolites when supplied with defined substrates. Such functional assays reveal whether a niche contains taxa capable of high-output sulfur metabolism, even if *in situ* measurements are influenced by host clearance, substrate availability, or other environmental constraints. Previous studies have demonstrated variability in amino acid degradation capacity among periodontal pathogens (18, 19) but have not extended this approach across multiple oral niches or related it to disease stage.

The immune response to oral biofilms is a central driver of periodontal tissue destruction (20, 21). Cytokines in subgingival crevice reflect the inflammatory status of the adjacent gingival tissue and influence microbial community structure through changes in nutrient availability, antimicrobial peptide expression, and tissue permeability. Certain cytokines, such as IL-1β, IL-6, and IL-17A, promote neutrophil recruitment and osteoclast activity (22, 23), while others, including IL-10 and IL-4, can dampen inflammatory damage and promote resolution. Mapping associations between cytokine profiles and microbial taxa can identify immune–microbe feedback loops that sustain either homeostasis or dysbiosis.

Despite this interlinked biology, sulfur metabolism is rarely examined in concert with local immune profiles and community structure. Investigations of subgingival sulfide have typically been separated from sequencing-based surveys of the microbiome, whereas cytokine studies often treat the biofilm as a black box (24, 25). Addressing these gaps demands a framework that treats VSCs as metabolic readouts, places them within the geography of the oral cavity, and links them to host inflammatory signals. Here, we integrate direct measurements of subgingival and oral-headspace VSCs with microbiome and cytokine profiling across five oral niches: saliva, tongue, subgingival, supragingival, and interdental plaque in cohorts representing clinical health, gingivitis, and chronic periodontitis. By mapping correlations among metabolite levels, community composition, and gingival crevicular fluid (GCF) cytokines, we test the premise that elevated subgingival sulfur amino acid degradation metabolites reflect a convergence of microbial functional capacity and inflammatory activation. We further evaluate whether sulfur-metabolic potential spreads beyond the periodontal pocket as disease advances, to better define functional dysbiosis in the oral ecosystem.

## MATERIALS AND METHODS

Participant recruitment and sample collection were performed through a case–control study of adults with health, gingivitis, or chronic periodontitis, recruited at Queen Mary University of London and the Royal London Dental Hospital (Barts Health NHS Trust) between February 2013 and May 2015 based on clinical criteria described previously (26). Eligibility required age 18–70 years and ≥20 teeth. Exclusions were systemic disease or respiratory infection, pregnancy or planning pregnancy, current smoking, recent antibiotics or anti-inflammatories (≤4 weeks), medications causing xerostomia, periodontal therapy in the prior 3 months, and denture wearing. Group assignment used full-mouth probing depth (PD) at six sites/tooth and bleeding on probing (BOP). Health—no caries/periodontitis; ≤4 pockets ≤4 mm (excluding third molars); BOP ≤20%. This group was stratified by plaque index (PI ≤30% “low plaque”; >30% “high plaque”). Gingivitis—generalized gingival inflammation, BOP >20%, PI >30%, and no generalized periodontal pocketing. Chronic periodontitis: radiographic bone loss, ≥2 sites with PD ≥6 mm (excluding third molars), and clinical attachment loss >2 mm.

### Sample collection

Participants refrained from eating and oral hygiene for ≥2 h (and malodorous foods for ≥12 h) before sampling. We collected oral-headspace, unstimulated saliva, tongue dorsum biofilm, subgingival plaque (deepest site in each quadrant; buccal and lingual), and interdental supragingival plaque (mesial/distal) using standard sterile techniques. Biofilm/plaque specimens were stabilized in TE buffer and stored at −80°C pending analysis. (Detailed devices, volumes, timings, and the sampling sequence appear in Supplemental methods). Oral-headspace volatile sulfur compounds (VSCs) were measured with a portable gas chromatograph (Oral Chroma CHM-1), as described previously (26). Participants sat upright and breathed nasally with mouths closed for ~1 min. Using a sterile mouthpiece and PTFE syringe positioned over the posterior tongue, ~15 mL of mouth air was withdrawn, and a 1 mL aliquot was injected for analysis.

### Cysteine and methionine degradation by headspace analysis

Protein in each specimen was quantified by a bicinchoninic acid assay. Aliquots (25 µL) of saliva, tongue, subgingival, supragingival, and interdental plaque were incubated at 37°C with gentle agitation in 10-mL headspace vials containing 0.5 mL of 0.5% (w/v) substrate sealed with an anaerobic headspace: cysteine (1 h) or methionine (24 h). Metabolism was stopped with addition of 1 mL ethanol, and headspace VSCs were quantified by gas chromatography with a sulfur-specific flame photometric detector (GC-FPD). Calibration used permeation-tube standards for H_2_S and CH_3_SH. Instrument configuration and run conditions are provided in Supplemental methods.

### Measurement of Subgingival VSCs

GCF-wetted paper strips were sealed in headspace vials, incubated at 80°C for 2 min, and 2 mL of headspace was injected into the GC-FPD as above. Standard curves reproduced the sampling workflow by dipping filter strips into serial dilutions of sodium sulfide or sodium thiomethoxide, sealing in vials, and acidifying with 1 M phosphoric acid before headspace sampling. VSC concentrations were interpolated from log–log calibration curves generated from known standards; these models also yielded limits of detection. Full device models, column/flow settings, and sampling-loop volumes are reported in Supplemental methods.

### Cytokine analysis

GCF for cytokine analysis was collected with sterile Whatman 3MM or Periocol strips, using two strips per tooth (buccal and lingual) as for subgingival VSC sampling. A 20-analyte panel of cytokines, chemokines, and adhesion molecules was quantified by multiplex bead immunoassay (FlowCytomix Human Inflammation 20-plex) and acquired on a BD FACSCanto II flow cytometer. GCF was eluted in TE, clarified by centrifugation, and stored at −80°C until analysis. Assays followed the manufacturer’s protocol: samples and standards were incubated with antibody-coupled beads, washed, labeled with streptavidin-PE, and read by flow cytometry. Five-parameter logistic (5-PL) standard curves were used for calibration, and concentrations were computed with the vendor software. Full reagent identifiers, volumes, incubation/wash conditions, and instrument settings are provided in Supplemental methods.

### DNA extraction

The dental plaque and tongue biofilm samples were disrupted from their respective sampling devices by transferring three to five sterile glass beads into the sample containing tubes and vortexing vigorously for 20 s. A 1-mL aliquot of the saliva sample was pelleted (>10,000 × *g*) for 10 min at 4°C, and the pellet homogenized in TE buffer. DNA extraction was carried out on all samples using the MasterPure Gram-positive DNA purification kit (Epicenter, Madison, USA), with an initial overnight incubation at 37°C with 1 µL MasterPure Ready-Lyse lysozyme per sample.

### Quantitative PCR assay

Absolute abundances of *Fusobacterium nucleatum*, *Tannerella forsythia*, *Aggregatibacter actinomycetemcomitans*, and *Porphyromonas gingivalis* were quantified by SYBR Green qPCR using published primers targeting *16S* or *rpoB* (27). Genomic DNA from pure cultures was used to generate standard curves, converting Ct values to CFU equivalents. Clinical DNA was diluted 1:10 to minimize inhibition. Reactions were run in duplicate with no-template controls and positive controls; specificity was confirmed by melt-curve analysis. Amplification was performed on a real-time PCR platform with standard cycling. A universal 16S-based primer set was used to estimate total bacterial load. For some analyses, species-specific abundances were also expressed as proportions relative to total 16S copies. Because such proportions depend on 16S copy-number variation and the breadth of the universal primer set (28), they were used only for normalization, not as absolute composition estimates. Supplemental methods provide primer sequences and concentrations, reaction mix, thermocycling program and details of standard preparation.

### Sequencing and bioinformatic analysis

Samples were screened for quality, and the bacterial 16S rRNA gene region V3-V4 was amplified using custom 341F-806R primers (29). Libraries were barcoded, multiplexed, and sequenced on MiSeq v2, 2 × 250 bp. Reads were processed with DADA2 (v1.24.0, R 4.3.0) (30): primers/adapters removed; forward/reverse trimmed to 230/210 bp; reads with maxEE >2 (forward) or >4 (reverse) discarded; maxN = 0. Error profiles were learned, reads denoised and merged, and chimeras removed (consensus). ASVs were assigned with eHOMD v15.1 and collapsed to the lowest confident rank to reduce sparsity. Count data were CLR-transformed for downstream analyses. Predicted metagenomes were generated with PICRUSt2 (defaults) and mapped to MetaCyc pathways (31). The raw sequence data are in the NCBI Sequence Read Archive (BioProject ID: PRJNA649803).

### Statistical analysis

All statistical analyses and data processing were conducted using R (v4.5.0). Cytokines were z-scored and summarized by PCA (FactoMineR). Microbiome abundances were CLR-transformed (pseudocount). Spearman correlations were computed among cytokines, taxa, and VSCs within and across niches/diagnoses; multiple testing was controlled by Benjamini–Hochberg (q < 0.05), and associations with |ρ| ≥ 0.4 were reported (psych). PICRUSt2 pathway abundances were merged with clinical metadata to examine sulfur-metabolism differences across niches and diagnoses. qPCR abundances were analyzed as absolute CFU equivalents and as proportions normalized to universal 16S, and related to amino acid–derived VSCs (raw and protein-normalized). Correlation networks were constructed using microbial sequencing data, GCF cytokines, and oral and subgingival VSC measurements. Because correlation-based networks capture both direct and indirect associations, they were interpreted as descriptive summaries of statistical relationships rather than conditional dependency structures. Full edge lists for all networks are provided in Data S1. Functional amino acid degradation assays were analyzed separately and were not included in the network correlation analyses. Networks were computed within each niche and diagnosis group using pairwise-complete observations, such that correlations were calculated using all available non-missing data for each variable pair rather than restricting analyses to complete cases across all assays. Clinically healthy participants were stratified into low-plaque and high-plaque where sample size allowed, otherwise combined as health. Multivariable linear regression models using the *lm* function from the base R stats package were fitted to evaluate associations between diagnosis and volatile sulfur compounds (oral headspace and GCF H_2_S and CH_3_SH) as well as GCF cytokines, adjusting for age, sex, and plaque index; models of VSCs additionally adjusted for subgingival bacterial load (qPCR-derived). Model diagnostics, including assessment of multicollinearity where applicable, were conducted using the *car* package. Outcomes were log-transformed as appropriate, and complete-case analysis was applied. Benjamini–Hochberg correction was used when multiple cytokines were tested. Visualizations used *pheatmap* and *ComplexHeatmap* (heatmaps/correlation matrices) and igraph/ggraph (networks), with variables classified as microbial, cytokine, or metabolite.

## RESULTS

In total, there were 92 participants, with microbial sequencing data available for 54 participants, GCF inflammatory cytokine data available for 45 participants, subgingival sulfide data available for 79 participants, amino acid degradation data for 32 participants, and qPCR microbial abundance data and intra-oral VSC concentrations available for all participants. Table 1 shows the demographic and clinical data in the study population. Overlap between clinical, sequencing, and cytokine data sets is illustrated in Fig. S1.

**TABLE 1:** Demographic and clinical data in the study population

Group (*n*)	Low plaque health (21)	High plaque health (20)	Gingivitis (26)	Periodontitis (25)
Mean age in years (SD)	34 ± 9.5	32.3 ± 7.3	34.4 ± 9.0	43.1 ± 7.4
Male:female	1.22	0.91	0.79	0.85
Mean plaque index (%)	18.0 ± 6.9	46.1 ± 13.6	46.3 ± 19.3	47.3 ± 22.9
Mean bleeding on probing (%)	7.8 ± 7.2	10.6 ± 6.2	24.6 ± 14.3	27.9 ± 23.5
Mean probing depths ≥ 4 mm (%)	1.3 ± 2.0	1.3 ± 1.4	6.1 ± 5.3	32.0 ± 17.8
Mean probing depths ≥ 5 mm (%)	0.6 ± 1.4	0.2 ± 0.4	1.6 ± 2.1	26.9 ± 17.5
Mean probing depths ≥ 6 mm (%)	0.1 ± 0.4	0 ± 0.1	0.2 ± 0.4	13.5 ± 13.9

The mean age was comparable across the health and gingivitis groups, ranging from 32.3 to 34.4 years, while participants in the periodontitis group were older, with a mean age of 43.1 ± 7.4 years. The male-to-female ratio was broadly balanced across all groups, ranging from 0.79 to 1.22. Clinical indices showed distinct patterns across diagnostic categories. The mean plaque index was lowest in the low plaque health group (18.0%, SD = 6.9), and substantially higher in the high plaque health (46.1%, SD = 13.6), gingivitis (46.3%, SD = 19.3), and periodontitis (47.3%, SD = 22.9) groups. Bleeding on probing increased progressively with disease severity: from 7.8% (SD = 7.2) in low plaque health to 27.9% (SD = 23.5) in periodontitis. Similarly, the extent of periodontal pocketing showed a clear gradient. In the periodontitis group, 32.0% (SD = 17.8) of sites had probing depths ≥4 mm, 26.9% (SD = 17.5) had depths ≥5 mm, and 13.5% (SD = 13.9) had depths ≥6 mm. In contrast, these values were markedly lower in all other groups, with minimal deep pocketing observed in the health and gingivitis groups.

### Sulfide levels and cytokine profiles mirror disease severity

**Fig 1:**
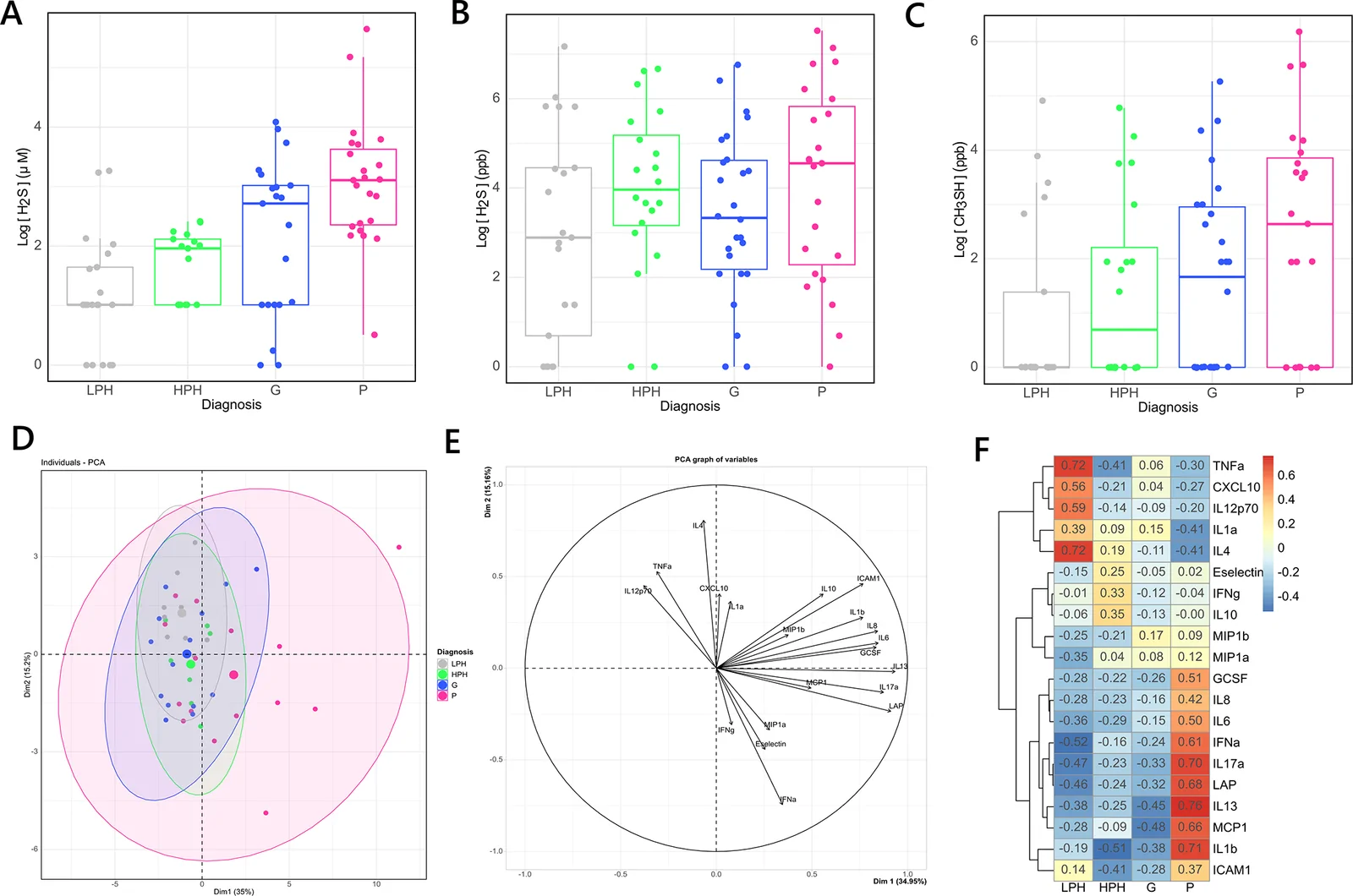
Relationship between subgingival, oral headspace VSCs, and cytokines in the gingival crevicular fluid. (**A**) Hydrogen sulfide concentrations detected in the gingival sulci of different diagnosis groups (LPH, low plaque health; HPH, high plaque health; G, gingivitis; P, periodontitis). (**B**) Hydrogen sulfide measured in the intra-oral headspace air of the different diagnosis groups. (**C**) Methanethiol measured in the intra-oral headspace air of the different diagnosis groups. (**D**) Score plot of the first two principal components in the principal component analysis of the 20 cytokines measured in the GCF samples of participants. (**E**) Loading plot of the PCA of cytokines showing different cytokine loadings. (**F**) Heatmap of the *z*-score standardized cytokine data, with row-wise mean *z*-score and cytokine clusters within different diagnosis groups shown.

Subgingival hydrogen sulfide (H_2_S) concentrations were significantly higher in gingivitis (mean = 17.8 µM) and periodontitis (mean = 37.5 µM) to low plaque health (mean = 7.49 µM), with a modest increase in high plaque health (Fig. 1A). In oral headspace air, periodontitis patients and individuals with high plaque scores exhibited higher H_2_S concentrations compared with the low plaque health group (Fig. 1B). Methanethiol concentrations were elevated in periodontitis, with gingivitis also showing higher levels than both health groups (Fig. 1C). In multivariable models adjusting for age, sex, plaque index, and subgingival bacterial load (determined by qPCR), periodontitis remained independently associated with elevated subgingival H_2_S (β = 1.23, *P* = 0.030). In contrast, oral headspace H_2_S did not retain independent association with diagnosis after adjustment. Oral methanethiol (CH_3_SH) was significantly associated with subgingival bacterial load (β = 0.40, *P* = 0.047) but not diagnosis, indicating that oral headspace methanethiol reflects subgingival microbial biomass (Table S3). Inclusion of salivary bacterial load in sensitivity analyses did not materially alter these findings.

Principal component analysis (PCA) of 20 cytokines in gingival crevicular fluid (GCF) revealed clustering of health and gingivitis profiles, while periodontitis exhibited more heterogeneous and distinct cytokine signatures (Fig. 1D). The corresponding heatmap of *z*-scored cytokine abundances (Fig. 1E) demonstrated elevated IL-4, CXCL10, IL-12p70, and tumor necrosis factor-alpha (TNFα) in low plaque health. In contrast, high plaque health was characterized by increased macrophage inflammatory protein 1-alpha (MIP-1α), E-selectin, interferon gamma (IFNγ), and IL-10, with IL-10 being among the most elevated cytokines overall. Gingivitis profiles showed reduced levels of cytokines enriched in high plaque health and upregulation of MIP-1α, macrophage inflammatory protein 1-alpha (MIP-1β), with decreased IL-13 and monocyte chemoattractant protein-1 (MCP-1). Periodontitis was associated with a broad inflammatory signature, with increased levels of IL-1β, IL-13, latency-associated peptide (LAP), IL-17A, interferon alpha (IFNα), IL-6, IL-8, and granulocyte-colony stimulating factor (G-CSF), and marked reductions in IL-4 and IL-1α. In multivariable models adjusting for age, sex, and plaque index, IL-6 (β = 2.35, *P* = 0.006), and IL-17A (β = 2.01, *P* = 0.029) were nominally associated with periodontitis. However, these associations did not remain significant after correction for multiple testing (Table S4).

### Functional sulfur metabolism and VSC production redistributes across niches in disease

**Fig 2:**
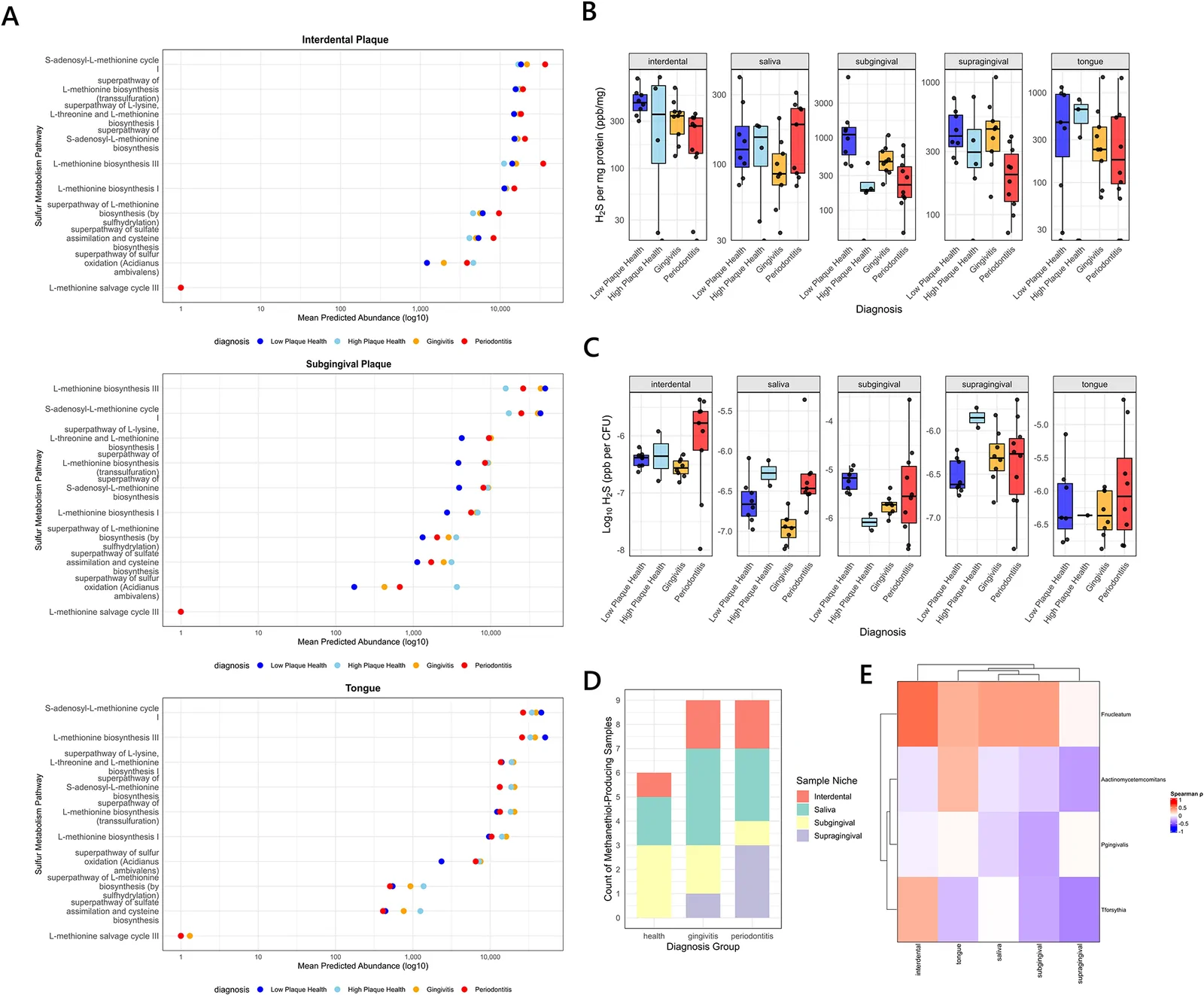
Microbial sulfur metabolism and cysteine/methionine degradation potential across oral niches and diagnostic groups. (**A**) Predicted functional abundance of sulfur-related metabolic pathways across subgingival, interdental, and tongue niches in health (low and high plaque), gingivitis, and periodontitis. Points represent mean pathway abundances for each group. Taxa contributing to predicted sulfur metabolism pathways are listed in Data S1. (**B**) Hydrogen sulfide (H_2_S) measured in the headspace of L-cysteine incubated samples from five oral niches, normalized to sample protein content (ppb/mg protein), highlighting variation in metabolic output relative to biomass. (**C**) The same H_2_S measurements normalized to qPCR estimated total bacterial abundance in the samples. (**D**) Number of samples demonstrating L-methionine degradation to methanethiol in each diagnosis group, with bar segments indicating contribution from each niche. (**E**) Heatmap showing Spearman correlation coefficients between relative abundance of select bacterial taxa as measured by qPCR and cysteine-derived H_2_S output across niches.

Predicted metabolic pathway abundances revealed a clear disease-linked redistribution of sulfur metabolism across niches. (Fig. 2A). The S-adenosyl-L-methionine cycle I and L-methionine biosynthesis pathways were most abundant in subgingival and tongue niches in low plaque health. In subgingival plaque, these pathways were reduced in high plaque health, whereas in the tongue, reductions were most pronounced in periodontitis; other sulfur-related pathways were also upregulated in diseased states. Methionine biosynthesis superpathways were particularly enriched in high plaque health and gingivitis cohorts in the tongue. In the cysteine incubation experiments, subgingival niche demonstrated the highest H_2_S production per mg protein in the samples, followed by supragingival niche and the tongue (Fig. 2B). Subgingival and supragingival samples from low plaque health and gingivitis cohorts produced higher normalized H_2_S than high plaque health and periodontitis. The higher output of subgingival and tongue samples was consistent, when the measured H_2_S was normalized to the total bacterial load as estimated by qPCR assays (Fig. 2C). Methanethiol detection following methionine incubation was most frequent in saliva samples (Fig. 2D). Gingivitis and periodontitis groups had more methionine-degrading samples than healthy controls. These findings, together with the pathway analysis, suggest distinct mechanisms for volatile production across niches, likely mediated by different microbial taxa. Analysis of absolute microbial abundance using qPCR data indicated that *Fusobacterium nucleatum* was consistently associated with cysteine degradation across most niches, highlighting its potential role as a key VSC producer *in vivo*.

### Disease-associated VSC variability reflects shifts in microbial community composition

**Fig 3:**
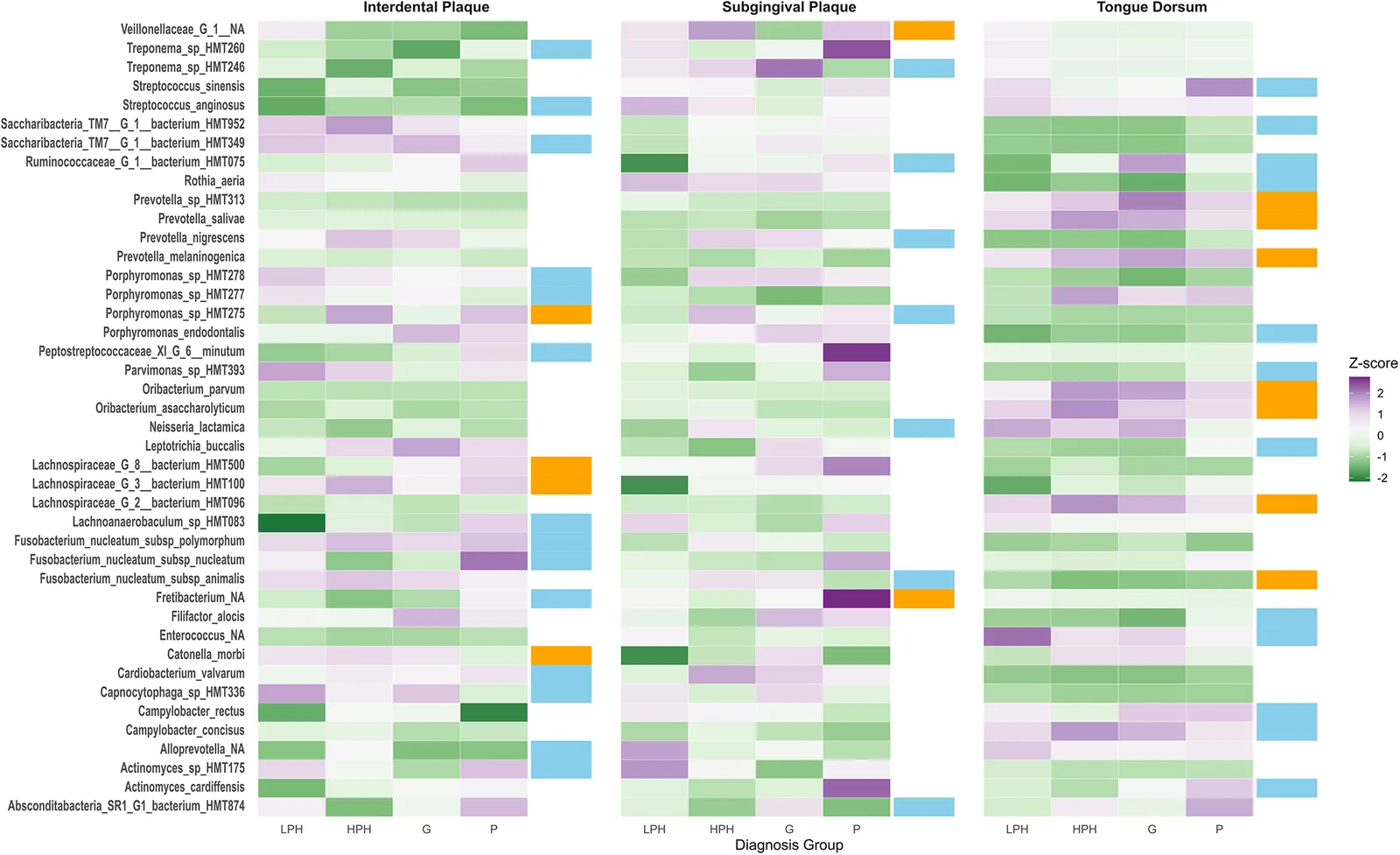
Heatmaps depicting the abundance of microbial taxa significantly correlated with subgingival volatile sulfur compound (VSC) concentrations across different oral niches and clinical diagnosis groups (LPH, low plaque health; HPH, high plaque health; G, gingivitis; P, periodontitis). Microbial taxa shown were identified based on their significant Spearman correlations (|ρ| > 0.4, FDR-adjusted *P* < 0.05) with gingival crevicular fluid (GCF) hydrogen sulfide or methanethiol levels, separately within the interdental, subgingival, and tongue niches. For visualization, centered log-ratio (CLR) transformed abundances of these taxa were *z*-score standardized across all samples to facilitate direct comparison across niches and diagnosis groups. A side bar for each niche indicates the correlation sign for each microbe-niche combination (orange = negative, blue = positive, white = none).

We next focused on microbial taxa that varied in association with subgingival VSC concentrations. Across niches, the interdental plaque and tongue showed more VSC-associated taxa than the subgingival plaque, suggesting broader community shifts in these sites. In interdental plaque, positive associations with subgingival VSCs were observed for *Fusobacterium nucleatum* subsp. *nucleatum*, *Fusobacterium nucleatum* subsp. *polymorphum*, *Fretibacterium* spp., *Streptococcus anginosus*, *Peptostreptococcaceae minutum*, *Catonella morbi*, *Porphyromonas* HMT 275, and *Lachnospiraceae* spp. were negatively associated with VSCs (Fig. 3A).

In subgingival plaque, *Prevotella nigrescens*, *Fusobacterium nucleatum* subsp. *animalis*, *Treponema* HMT 246, *Neisseria lactamica*, and *Ruminococcaceae [G-1]* HMT075 were positively associated with subgingival VSCs. The tongue microbiota showed positive associations between subgingival VSCs and several periodontitis-associated taxa, including *Porphyromonas endodontalis*, *Filifactor alocis*, *Campylobacter rectus*, *Campylobacter concisus*,* Enterococcus* spp., and *Streptococcus sinensis*. Within the periodontitis cohort, *Prevotella* such as *P. melaninogenica*, *P. salivae*, *P.* HMT 313, and *Oribacterium* such as *O. parvum* and *O. asaccharolyticum*, were negatively associated with subgingival VSCs.

### How do host and microbes interact? cytokine–microbe correlations reveal niche-specific immune responses

**Fig 4:**
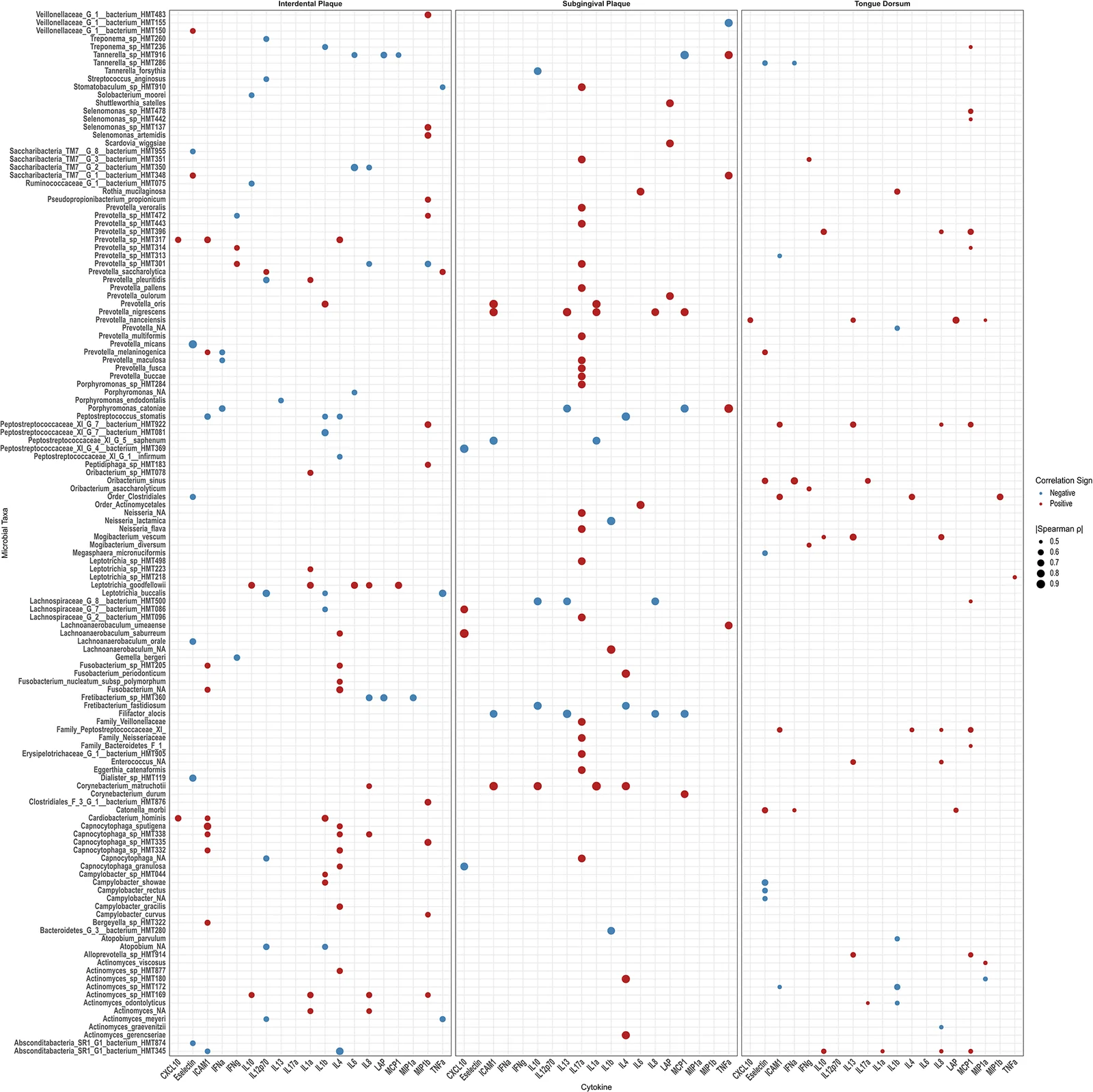
Microbe-cytokine correlation dot plot across oral niches in individuals without periodontitis. Significant correlations (Spearman |ρ| > 0.4, FDR-adjusted *P* < 0.05) between microbial taxa and cytokine abundances were determined separately within the interdental, subgingival, and tongue niches, excluding periodontitis patients to focus on early dysbiotic and/or health-associated interactions. Centered log-ratio (CLR) transformed microbial data and *z*-score standardized cytokine data were used. Microbe-cytokine correlations heatmaps for periodontitis.

Associations between microbial taxa and cytokine levels were investigated to map immune-microbial interactions within each niche. In interdental plaque, *Capnocytophaga* spp. and *Fusobacterium* spp. were positively correlated with ICAM1 and IL-4 levels (Fig. 4A), while *Prevotella* spp. showed negative correlations with IFNα. Notably, *Prevotella oris* was positively associated with IL-1β, and *Selenomonas* spp. were correlated with MIP-1β. In subgingival plaque, *Corynebacterium matruchotii* positively correlated with multiple cytokines (ICAM1, IL-10, IL-1α, IL-4), and *Prevotella nigrescens* showed strong positive associations with ICAM1, IL-13, IL-1α, IL-8, and MCP-1. In contrast, *Filifactor alocis* was negatively associated with these same cytokines. IL-17A was associated with multiple *Prevotella* species. *Tannerella* HMT916 was negatively associated with MCP-1 across niches, and *T. forsythia* with IL-10 in subgingival plaque. Other notable associations included *Scardovia wiggsiae* and *Shuttleworthia satelles* with LAP, and *Rothia mucilaginosa* with IL-6. In the tongue, *Campylobacter rectus, C. showae,* and *Megasphaera micronuciformis* negatively correlated with E-selectin, while *P. melaninogenica* was positively associated. *R. mucilaginosa* again correlated positively with IL-1β, and *Mogibacterium* spp. in the tongue were positively associated with IL-13, IL-10, IL-8, and IFNα (Fig. 4C).

### Correlation networks reveal shifts in cytokine-microbe-VSC associations across disease states

**Fig 5:**
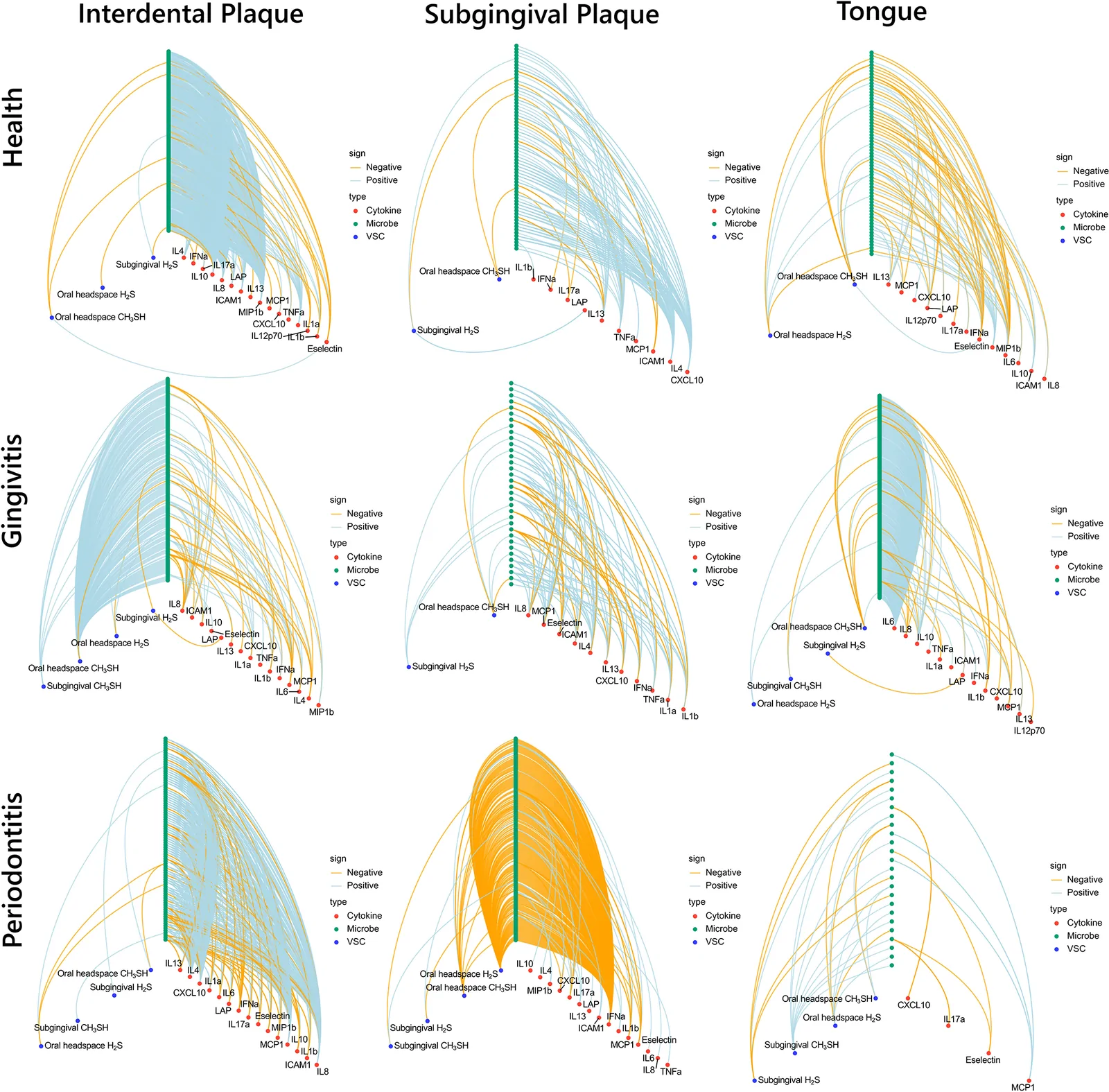
Hive plots representing significant associations between microbial taxa, inflammatory cytokines, and volatile sulfur compounds (VSCs) across oral niches and disease states. The network graphs visualize significant spearman correlations (|ρ| > 0.4, FDR-adjusted *P* < 0.05) detected between bacterial taxa (green nodes), cytokines (orange nodes), and VSC concentrations (pink nodes) across three oral niches (interdental, subgingival, tongue) arranged as columns and three diagnostic categories (health, gingivitis, periodontitis) arranged as rows in the panel. Edges represent positive or negative correlations scaled by correlation strength. Microbial node labels are omitted for clarity, while subgingival and oral headspace concentrations of hydrogen sulfide (H_2_S) and methanethiol (CH_3_SH) are included as VSC nodes. All the plotted edges and their strength of association are provided in the Data S2.

Spearman correlation networks revealed distinct interaction patterns between microbial taxa, cytokines, and volatile sulfur compounds (VSCs) across oral niches and diagnostic categories. In health, dental plaque niches (interdental and subgingival) showed predominantly negative associations between cytokines and microbial taxa, whereas the tongue displayed more positive associations (Fig. 5, top row). Gingivitis was associated with a reduced number of total associations, but more of these were positive in direction. Notably, GCF IL-6 showed numerous positive associations with tongue-associated microbes in gingivitis (Fig. 5, middle row). In periodontitis, the increased microbial and cytokine complexity led to a greater number of significant associations, particularly within periodontal niches. These included positive cytokine–microbe associations in the interdental niche and negative associations in the subgingival plaque, suggesting niche-specific immune-microbiota interactions (Fig. 5, bottom row). IL-4 and IL-8 were positively associated with multiple taxa in the interdental plaque of periodontitis patients, whereas IFNα, CXCL10, and IL-17A showed predominantly negative associations. VSCs in oral headspace were more commonly associated with microbes in periodontal niches in both gingivitis and periodontitis. However, the strongest microbial associations with subgingival VSCs were found in the tongue (periodontitis) and interdental plaque (gingivitis), indicating cross-niche interactions.

## DISCUSSION

This study delivers a multi-omic view of volatile sulfur compound (VSC) production and host–microbial interactions across five oral niches spanning health, gingivitis, and periodontitis. Integrating functional predictions, metabolite quantification, cytokine profiling, and correlation networks, we show that inflammatory programs, community composition, and sulfur metabolic potential vary by niche and disease state, with the subgingival niche strongly linked to oral-headspace VSCs.

### Volatile sulfur compound dynamics reflect disease and niche-ecology

Subgingival H_2_S concentrations exhibited a clear progressive increase from low plaque health (LPH), through high plaque health (HPH) and gingivitis, to periodontitis (Fig. 1A). This pattern was mirrored by methanethiol concentrations measured in the oral headspace, suggesting that these two VSCs may reflect disease progression more robustly than oral headspace H_2_S, which showed a bimodal distribution (Fig. 1B). Notably, oral headspace H_2_S was elevated in both HPH and periodontitis but lower in LPH and gingivitis, a pattern that closely paralleled the previously described subgingival plaque Shannon diversity in these cohorts (26). The bimodal distribution of oral headspace H_2_S observed in individuals with high plaque health and periodontitis likely reflects distinct but convergent ecological states that support sulfide accumulation via different mechanisms. In high plaque health, elevated H_2_S may arise from increased total microbial biomass and community diversity, particularly on the tongue and supragingival surfaces, where generalist and facultative anaerobes contribute cumulatively to cysteine degradation despite limited overt inflammation. In contrast, the elevated H_2_S observed in periodontitis is more plausibly driven by disease-associated ecological restructuring, characterized by expansion of obligate anaerobes, enhanced proteolytic activity, and increased availability of sulfur-containing substrates derived from host inflammation and tissue breakdown. Thus, while both states yield elevated oral headspace H_2_S, they likely do so through fundamentally different ecological routes: biomass- and diversity-driven metabolism in high plaque health versus inflammation-coupled, functionally specialized sulfur metabolism in periodontitis. The lower oral headspace H_2_S levels observed in gingivitis may represent a transitional state in which inflammatory substrate availability and microbial metabolic specialization have not yet fully aligned. These trends raise the possibility that microbial richness and evenness within the subgingival niche contribute to oral H_2_S generation in a context-dependent manner, rather than through a single disease-linked mechanism. This interpretation is supported by the elevated H_2_S production per mg protein observed in subgingival plaque, indicating that sulfide output may reflect both community structure and functional capacity. Several periodontal pathogens harbor methionine γ-lyase (*mgl*), an enzyme capable of degrading both methionine and cysteine, suggesting a potential mechanistic link between disease-associated microbial enrichment and sulfide production (6, 32). Accordingly, the elevated H_2_S observed in periodontitis may arise from increased representation of *mgl*-positive taxa alongside greater availability of sulfur-containing substrates driven by inflammation and tissue breakdown. Together, these findings suggest that, in addition to saliva, the subgingival plaque can contribute appreciably to the oral headspace VSC pool, with its impact shaped by biofilm biomass, community composition, and inflammatory state.

### Functional insights from sulfur metabolism pathways and VSC production assays

Methionine degradation capacity was detected more frequently in gingivitis and periodontitis samples, particularly from interdental and supragingival niches, whereas in health, this capability was more restricted to subgingival plaque (Fig. 2D). This suggests a disease-associated expansion of functional niches capable of methanethiol generation. The pathway analysis corroborated this, highlighting pronounced differences in sulfur metabolism pathways across diagnosis groups (Fig. 2A). Subgingival plaque exhibited marked enrichment of cysteine and methionine degradation pathways in disease, while pathways like S-adenosyl-L-methionine cycle and methionine biosynthesis were more prevalent in health. Cysteine degradation results mirrored trends observed in oral headspace H_2_S and Shannon diversity; subgingival and supragingival H_2_S production was higher in LPH and gingivitis, and lower in HPH and periodontitis (Fig. 2B and 2C). It is conceivable that as periodontal disease progresses, structural and immunological changes in biofilms may reduce the per-unit-protein VSC output despite increased total abundance, reflecting a trade-off in community function. One hypothesis is that high-diversity communities in health (high plaque) and periodontitis may contain more generalist taxa with distributed sulfur metabolism, whereas more specialized producers dominate in LPH and gingivitis. Alternatively, local environmental constraints, such as host protease activity or nutrient flux, may modulate enzymatic activity despite similar taxonomic profiles. Host redox chemistry may also influence detectable sulfide levels. Periodontal inflammation is associated with increased concentrations of host antioxidants such as glutathione, which can react with hydrogen sulfide and other reactive sulfur species. Such host-mediated detoxification or oxidation of sulfide could reduce measurable H_2_S output per unit biomass despite increased microbial sulfur metabolism potential in diseased sites. Although predicted abundances of methionine γ-lyase and related carbon–sulfur lyases were detected across niches, increasing abundance in the subgingival niche in disease, correlations with oral headspace CH_3_SH were weak. This likely reflects the fact that PICRUSt2 predicts functional potential rather than gene expression, and that VSC production arises from multiple microbial pathways and environmental conditions.

### Subgingival VSCs as a proxy for disease-associated microbial shifts across niches

Subgingival VSC concentrations were closely correlated with specific microbial taxa across niches and diagnosis groups. For example, *Fusobacterium nucleatum*, *Fretibacterium* spp., and *Porphyromonas endodontalis* were frequently associated with higher VSCs, particularly in interdental and tongue niches (Fig. 3). These taxa are established anaerobes with known cysteine desulfhydrase activity and have been consistently linked to periodontitis (16, 33). In contrast, *Catonella morbi* was negatively associated with VSCs, particularly in low plaque health, suggesting a possible antagonistic or protective role. These findings suggest that VSC generation is not limited to the site of measurement but may also reflect broader ecological shifts and inter-niche signaling as discussed above for the cytokine correlations. Importantly, we observed context-dependent associations, such as *Prevotella nigrescens*, which correlated with subgingival VSCs only in HPH and gingivitis, but not in periodontitis (Fig. 3). This suggests that specific taxa may participate in VSC generation at early disease stages but are replaced or outcompeted by more efficient sulfur metabolizers in advanced disease, or their spatial co-aggregation partners may change in different contexts which may lead to more antagonistic rather than synergistic metabolic interactions.

On the tongue, subgingival VSC concentrations were positively associated with several taxa conventionally implicated in periodontitis, including *Porphyromonas endodontalis, Filifactor alocis, Campylobacter rectus, Campylobacter concisus, Enterococcus* spp., and *Streptococcus sinensis*. These associations reinforce the emerging view of the tongue as a reservoir for periodontopathogens and as a potential sentinel for shifts occurring in subgingival ecology (34). Notably, such associations occurred despite the spatial separation between the tongue and subgingival niches, highlighting possible cross-niche microbial seeding or systemic factors influencing both habitats. The tongue dorsum provides a large, structured surface capable of sustaining anaerobic and facultative taxa, supporting its role as a reservoir for periodontopathogens and a source of cross-niche redistribution via saliva. The consistent association of tongue taxa with subgingival VSCs suggests that tongue community shifts may mirror, or in some cases precede, subgingival dysbiosis, highlighting the role of the tongue in periodontal ecological change. In subgingival plaque, strong positive correlations with subgingival VSCs were observed for *Prevotella nigrescens*, *Fusobacterium nucleatum* subsp. *animalis*, *Treponema* HMT 246, *Neisseria lactamica*, and *Ruminococcaceae* [G-1] HMT075, organisms with known or suspected roles in sulfur metabolism and periodontal dysbiosis. The presence of *N. lactamica*, typically considered a commensal, may reflect opportunistic shifts in community structure during disease progression. Interestingly, while several *Prevotella* and *Oribacterium* species (*P. melaninogenica*, *P. salivae*, *P.* HMT 313, *O. parvum*, *O. asaccharolyticum*) were enriched in periodontitis, they were negatively associated with subgingival VSCs, suggesting either a compensatory ecological role, competitive exclusion, or distinct metabolic contributions that do not support VSC accumulation (Fig. 3). In the low plaque health (LPH) group, subgingival VSCs were still linked to *Enterococcus* spp. and *F. nucleatum* subsp. *animalis*, suggesting that even in clinically healthy or low-inflammation environments, there may be early evidence of VSC-producing microbial activity, possibly primed by niche conditions rather than overt disease. Together, these findings support a model in which subgingival VSCs reflect the metabolic interaction of a dynamic and niche-specific microbial community, modulated by both health status and microbial composition. Rather than being mere byproducts of metabolism, VSCs may represent functional biomarkers of ecological shifts, with potential roles in shaping, or at least mirroring, host–microbe interactions across disease stages.

Taken together, these patterns suggest that *Prevotella* species occupy a context-dependent ecological role in sulfur metabolism and immune-associated dysbiosis. In early or transitional states, such as high plaque health and gingivitis, *Prevotella* may contribute to VSC production through cooperative metabolic interactions within mixed anaerobic consortia, potentially amplifying local inflammatory processes. In established periodontitis, however, the negative associations between several *Prevotella* species and subgingival VSCs imply either functional displacement by more specialized sulfur-metabolizing taxa or a shift toward compensatory metabolic roles that do not promote sulfide accumulation. Thus, rather than acting as uniform drivers of VSC production or inflammation, *Prevotella* spp. appear to function as flexible ecological participants whose metabolic and immunological relevance is tightly coupled to niche conditions and disease stage.

### Divergent inflammatory milieu across disease groups

Distinct cytokine expression profiles characterized each diagnostic group, reinforcing the view that periodontal disease progression is underpinned by dynamic shifts in host immune signaling. Healthy individuals with low plaque coverage (LPH) exhibited elevated levels of cytokines typically associated with homeostatic regulation and Th1-type responses, such as IL-4, TNFα, CXCL10, and IL-12p70 (Fig. 1D through F). Some of these cytokines have been previously implicated in protective mucosal immunity and are known to suppress inflammatory tissue damage (35). Interestingly, participants with high plaque coverage but clinically healthy gingiva (HPH) exhibited a distinct cytokine signature, with increased levels of MIP-1α, E-selectin, IFNγ, and IL-10, among others. These findings suggest that in the absence of overt inflammation, chronic plaque accumulation may still provoke a subclinical immune modulation skewed towards chemokine-mediated leukocyte recruitment and regulatory feedback, particularly via IL-10. This supports the concept of clinical health in the presence of “incipient microbial dysbiosis” proposed in contemporary models of periodontal disease (36).

In gingivitis, a transitionary state between health and periodontitis, the cytokine environment was characterized by upregulation of MIP-1α and MIP-1β, with concurrent suppression of IL-13 and MCP-1. The predominance of monocyte and macrophage chemoattractants is consistent with established lesion development and innate immune recruitment, while reduced IL-13 may signify impaired resolution of inflammation (37). By contrast, periodontitis samples exhibited a profoundly altered cytokine milieu, with marked upregulation of IL-1β, IL-6, IL-8, IL-17A, IL-13, G-CSF, IFNα, and MCP-1. These mediators represent a robust proinflammatory and Th17-dominated axis known to facilitate neutrophil influx, osteoclastogenesis, and connective tissue breakdown (22, 23). The suppression of IL-4 and IL-1α in this group further highlights the loss of regulatory counterbalance, consistent with a destructive inflammatory cascade.

### Cytokine-microbe networks reveal immune-microbial crosstalk in distinct niches

The cytokine-microbe-VSC correlation analysis (Fig. 5) revealed key insights into niche-specific microbe-cytokine interactions. In general, most cytokine-microbe correlations occurred within the interdental and subgingival niches, which aligns with the anatomical proximity of these communities to the GCF source of cytokines. Interestingly, the tongue showed a high volume of correlations in gingivitis, particularly involving IL6. This may suggest a tongue-specific inflammatory feedback loop in gingivitis, or compensatory microbial responses to low-grade inflammation. This finding is intriguing, as IL-6 is a known driver of epithelial barrier modulation and neutrophil activation, and its presence in the tongue milieu may reflect an early response to community shifts (23, 38). Similar tongue–cytokine associations have been reported in studies linking the salivary microbiota (which is similar to the tongue microbiota) with markers of inflammation in oral cancer (39). The broader network of cytokine-microbe associations across niches suggests that dominant cytokines within each disease group may not exclusively drive microbial interactions. For example, while IL6 emerged as a key correlate in gingivitis, other signals such as IL10, E-selectin, and MIP1b also influenced microbial dynamics, particularly on the tongue and in health—but do not form a distinguishing cluster in the PCA analysis (Fig. 1). This complex immuno-microbial interplay highlights the need to understand inflammatory signals as niche-modulated rather than systemically uniform across the oral cavity. In health, positive correlations between tongue microbes and cytokines such as E-selectin and MIP-1β suggest a more regulated immune–microbial interface. In contrast, in periodontitis, cytokine–microbe associations were predominantly observed in the subgingival and interdental niches, where the highest concentrations of GCF cytokines are encountered. Here, cytokines, such as IL-17A, IL-1β, and MCP-1, exhibited numerous associations with disease-associated taxa, supporting the hypothesis that local inflammation directly influences microbial ecology (20).

In interdental plaque, *Capnocytophaga* spp. and *Fusobacterium* spp. demonstrated consistent positive associations with ICAM-1 and IL-4 (Fig. 4). *Capnocytophaga* has been historically associated with health or disease depending on the niche it is found in, and its association with ICAM-1 and IL-4, a cytokine known to inhibit macrophage activation, may reflect a regulatory environment that maintains immune tolerance in the interdental niche (15, 40). Conversely, *Prevotella* spp. in this niche showed negative correlations with IFNα, suggesting that this genus may antagonize antiviral or type I interferon responses, consistent with their enrichment in anaerobic, low-IFN environments (41). *Prevotella oris* was positively associated with IL-1β in the same niche, indicating its potential role in pro-inflammatory recruitment and neutrophil activation.

In subgingival plaque, a greater breadth of immune interactions was evident. *Corynebacterium matruchotii*, a filamentous actinomycetota associated with dental health and plaque structural scaffolding, was positively correlated with ICAM-1, IL-10, IL-1α, and IL-4 (42). These correlations suggest that *C. matruchotii* may participate in maintaining immune equilibrium in plaque biofilms, or that plaque biofilm structure may be disrupted in conditions such as gingivitis and periodontitis. In contrast, *Prevotella nigrescens* demonstrated strong positive correlations with IL-13, IL-1α, IL-8, and MCP-1—cytokines consistently elevated in periodontitis, indicating its role in disease-associated inflammation. Notably, *Filifactor alocis*, a keystone pathogen increasingly recognized for its resistance to oxidative stress and pro-inflammatory capacity (43), exhibited negative associations with these same cytokines, suggesting niche competition or divergent immune-modulatory strategies (Fig. 4). Additionally, IL-17A, a cytokine important in neutrophilic inflammation, was associated with multiple *Prevotella* species, reinforcing the genus’s link to Th17 responses in periodontitis (44).

*Tannerella forsythia* showed a negative association with IL-10, a key anti-inflammatory cytokine, further supporting its role in promoting immune dysregulation (Fig. 4). Similarly, *Tannerella* HMT916 was negatively associated with MCP-1 across niches, suggesting a suppressive effect on monocyte recruitment. *Scardovia wiggsiae* and *Shuttleworthia satelles*, less frequently discussed in periodontal literature, showed positive correlations with LAP (latency-associated peptide of TGFβ), potentially linking them to matrix remodeling or fibrosis. *Rothia mucilaginosa,* an early colonizer with debated pathogenicity, correlated positively with IL-6 in subgingival plaque and IL-1β in the tongue—both cytokines reflective of pro-inflammatory responses. It has been recognized that *R. mucilaginosa* exhibits strong niche-specific colonization, with high prevalence and relative abundance particularly in the tongue dorsum and upper respiratory tract, where it is considered part of the commensal core microbiota. As a mucosa-associated, microaerophilic generalist, *R. mucilaginosa* is well adapted to oxygen and nutrient gradients at epithelial surfaces rather than to the deeply anaerobic conditions characteristic of active sulfur metabolism. However, interpreting these cytokine associations is not straightforward. Given its dominance in the tongue microbiota, the observed positive correlation with IL-1β may reflect community-level shifts rather than specific immune activation by *R. mucilaginosa* itself. As cytokine levels and microbial community structure both vary along the disease continuum, from health through gingivitis to periodontitis, many such correlations may be driven by co-occurring changes rather than causal interactions. In other words, *R. mucilaginosa*’s association with inflammatory cytokines could be a consequence of broader ecological perturbations, rather than direct immunomodulatory activity. Furthermore, in niches like the subgingival plaque, where *Rothia* species are less dominant but still detectable, the observed IL-6 correlation may represent a localized ecological response or reflect spill-over effects from tongue-associated microbial shifts, especially if cross-niche interactions influence community assembly and host response. These uncertainties highlight an important limitation of correlation-based network analyses: they do not disentangle direct microbial influence on host signaling from indirect associations linked to community restructuring. Thus, while intriguing, the *Rothia*-cytokine correlations should be interpreted with caution, and ideally validated through mechanistic or longitudinal studies to clarify their biological significance. Viewed in this light, *R. mucilaginosa* may function less as an active driver of inflammation and more as a niche-adapted responder whose abundance and immune associations reflect the prevailing inflammatory processes of the local environment, particularly at mucosal and microaerophilic niches. Such taxa may therefore serve as indicators or stabilizing components of community restructuring across oral niches, rather than direct mediators of tissue-destructive immune responses.

In the tongue, *Campylobacter* spp., particularly *C. rectus* and *C. showae*, negatively correlated with E-selectin, a key adhesion molecule involved in neutrophil rolling and transendothelial migration (Fig. 4). The functional implications of this remain speculative but may indicate suppression of endothelial activation. Conversely, *Prevotella melaninogenica* was positively associated with E-selectin, suggesting differential contributions to epithelial inflammation. *Mogibacterium* spp. also stood out in the tongue microbiome, showing broad positive associations with IL-13, IL-10, IL-8, and IFNα, suggesting that this group may interact with both pro- and anti-inflammatory pathways depending on local context.

### Conclusion

Together, these findings demonstrate a nuanced and spatially stratified relationship between microbial community composition, inflammatory cytokine signaling, and sulfur metabolism within the oral cavity. By integrating molecular, functional, and ecological approaches, we find that periodontal niches, particularly the subgingival and interdental sites, are central hubs of host–microbial interaction and VSC metabolism, with disease progression associated with expanded metabolic potential and broader microbial engagement. However, the contribution of periodontal niches to whole-mouth malodor remains dependent on relative biomass and protein content, with saliva continuing to dominate in absolute terms. The distinct cytokine profiles and sulfur metabolic pathways identified here offer new insights into the pathophysiology of periodontal disease and raise the possibility that VSCs, particularly subgingival H_2_S, may serve as functional biomarkers of dysbiosis and inflammation. Further longitudinal studies and metatranscriptomic analyses will be required to validate these pathways and assess their utility in clinical diagnostics and therapeutic targeting.

## Data Availability

Raw sequencing data have been deposited in the NCBI Sequence Read Archive (SRA) under BioProject accession PRJNA649803. All processed datasets, including clinical metadata, volatile sulfur compound measurements, cytokine data, qPCR data, PICRUSt2 functional prediction outputs, supplemental data sets, and the R scripts used to reproduce all analyses and figures are publicly available at https://github.com/AbishStephen/oral-sulfur-metabolism-microbiome.

## SUPPLEMENTAL MATERIAL

Data S1 spectrum.03008-25-s0001.csv Taxa contribution to predicted sulfur pathways.

Data S2 spectrum.03008-25-s0002.csv Full edge list as plotted in the network analyses in Figure 5.

Supplemental methods spectrum.03008-25-s0003.docx Additional methods.

ASM does not own the copyrights to Supplemental Material that may be linked to, or accessed through, an article. The authors have granted ASM a non-exclusive, world-wide license to publish the Supplemental Material files. Please contact the corresponding author directly for reuse.

## References

[1] Scully C, Greenman J. 2008. Halitosis (breath odor). Periodontol 2000 48:66–75. doi:10.1111/j.1600-0757.2008.00266.x 18715357

[2] Greenman J, Duffield J, Spencer P, Rosenberg M, Corry D, Saad S, Lenton P, Majerus G, Nachnani S, El-Maaytah M. 2004. Study on the organoleptic intensity scale for measuring oral malodor. J Dent Res 83:81–85. doi:10.1177/154405910408300116 14691119

[3] Bollen CML, Beikler T. 2012. Halitosis: the multidisciplinary approach. Int J Oral Sci 4:55–63. doi:10.1038/ijos.2012.39 PMC3412664 22722640

[4] Persson S, Edlund M-B, Claesson R, Carlsson J. 1990. The formation of hydrogen sulfide and methyl mercaptan by oral bacteria. Oral Microbiol Immunol 5:195–201. doi:10.1111/j.1399-302x.1990.tb00645.x 2082242

[5] Stephen AS, Naughton DP, Pizzey RL, Bradshaw DJ, Burnett GR. 2014. *In vitro* growth characteristics and volatile sulfur compound production of *Solobacterium moorei*. Anaerobe 26:53–57. doi:10.1016/j.anaerobe.2014.01.007 24487184

[6] Stephen AS, Millhouse E, Sherry L, Aduse-Opoku J, Culshaw S, Ramage G, Bradshaw DJ, Burnett GR, Allaker RP. 2016. *In vitro* effect of *Porphyromonas gingivalis* methionine gamma lyase on biofilm composition and oral inflammatory response. PLoS One 11:e0169157. doi:10.1371/journal.pone.0169157 PMC5199072 28033374

[7] Allaker RP, Waite RD, Hickling J, North M, McNab R, Bosma MP, Hughes FJ. 2008. Topographic distribution of bacteria associated with oral malodour on the tongue. Arch Oral Biol 53:S8–S12. doi:10.1016/S0003-9969(08)70003-7 18460402

[8] Persson S. 1992. Hydrogen sulfide and methyl mercaptan in periodontal pockets. Oral Microbiol Immunol 7:378–379. doi:10.1111/j.1399-302x.1992.tb00641.x 1299802

[9] Morita M, Wang HL. 2001. Relationship between sulcular sulfide level and oral malodor in subjects with periodontal disease. J Periodontol 72:79–84. doi:10.1902/jop.2001.72.1.79 11210077

[10] Torresyap G, Haffajee AD, Uzel NG, Socransky SS. 2003. Relationship between periodontal pocket sulfide levels and subgingival species. J Clin Periodontol 30:1003–1010. doi:10.1034/j.1600-051x.2003.00377.x 14761124

[11] Yaegaki K, Sanada K. 1992. Volatile sulfur compounds in mouth air from clinically healthy subjects and patients with periodontal disease. J Periodontal Res 27:233–238. doi:10.1111/j.1600-0765.1992.tb01673.x 1640345

[12] Ratcliff PA, Johnson PW. 1999. The relationship between oral malodor, gingivitis, and periodontitis. A review. J Periodontol 70:485–489. doi:10.1902/jop.1999.70.5.485 10368052

[13] De Geest S, Laleman I, Teughels W, Dekeyser C, Quirynen M. 2016. Periodontal diseases as a source of halitosis: a review of the evidence and treatment approaches for dentists and dental hygienists. Periodontol 2000 71:213–227. doi:10.1111/prd.12111 27045438

[14] Tang‐Larsen J, Claesson R, Edlund M‐B, Carlsson J. 1995. Competition for peptides and amino acids among periodontal bacteria. J Periodontal Res 30:390–395. doi:10.1111/j.1600-0765.1995.tb01292.x 8544102

[15] Socransky SS, Haffajee AD, Cugini MA, Smith C, Kent RL Jr. 1998. Microbial complexes in subgingival plaque. J Clin Periodontol 25:134–144. doi:10.1111/j.1600-051x.1998.tb02419.x 9495612

[16] Colombo APV, Boches SK, Cotton SL, Goodson JM, Kent R, Haffajee AD, Socransky SS, Hasturk H, Van Dyke TE, Dewhirst F, Paster BJ. 2009. Comparisons of subgingival microbial profiles of refractory periodontitis, severe periodontitis, and periodontal health using the human oral microbe identification microarray. J Periodontol 80:1421–1432. doi:10.1902/jop.2009.090185 PMC3627366 19722792

[17] Yaegaki K. 2008. Oral malodorous compounds are periodontally pathogenic and carcinogenic. Jpn Dent Sci Rev 44:100–108. doi:10.1016/j.jdsr.2008.06.003

[18] Persson S, Claesson R, Carlsson J. 1989. The capacity of subgingival microbiotas to produce volatile sulfur compounds in human serum. Oral Microbiol Immunol 4:169–172. doi:10.1111/j.1399-302x.1989.tb00246.x 2639302

[19] Basic A, Dahlén G. 2015. Hydrogen sulfide production from subgingival plaque samples. Anaerobe 35:21–27. doi:10.1016/j.anaerobe.2014.09.017 25280920

[20] Hajishengallis G. 2014. Immunomicrobial pathogenesis of periodontitis: keystones, pathobionts, and host response. Trends Immunol 35:3–11. doi:10.1016/j.it.2013.09.001 PMC3947349 24269668

[21] Hajishengallis G, Korostoff JM. 2017. Revisiting the Page & Schroeder model: the good, the bad and the unknowns in the periodontal host response 40 years later. Periodontol 2000 75:116–151. doi:10.1111/prd.12181 PMC5539911 28758305

[22] Gaffen SL, Hajishengallis G. 2008. A new inflammatory cytokine on the block: re-thinking periodontal disease and the Th1/Th2 paradigm in the context of Th17 cells and IL-17. J Dent Res 87:817–828. doi:10.1177/154405910808700908 PMC2692983 18719207

[23] Dutzan N, Kajikawa T, Abusleme L, Greenwell-Wild T, Zuazo CE, Ikeuchi T, Brenchley L, Abe T, Hurabielle C, Martin D, Morell RJ, Freeman AF, Lazarevic V, Trinchieri G, Diaz PI, Holland SM, Belkaid Y, Hajishengallis G, Moutsopoulos NM. 2018. A dysbiotic microbiome triggers T_H_17 cells to mediate oral mucosal immunopathology in mice and humans. Sci Transl Med 10:eaat0797. doi:10.1126/scitranslmed.aat0797 PMC6330016 30333238

[24] Thunell DH, Tymkiw KD, Johnson GK, Joly S, Burnell KK, Cavanaugh JE, Brogden KA, Guthmiller JM. 2010. A multiplex immunoassay demonstrates reductions in gingival crevicular fluid cytokines following initial periodontal therapy. J Periodontal Res 45:148–152. doi:10.1111/j.1600-0765.2009.01204.x PMC5405696 19602112

[25] Bamashmous S, Kotsakis GA, Kerns KA, Leroux BG, Zenobia C, Chen D, Trivedi HM, McLean JS, Darveau RP. 2021. Human variation in gingival inflammation. Proc Natl Acad Sci USA 118:1–10. doi:10.1073/pnas.2012578118 PMC8271746 34193520

[26] Stephen AS, Dhadwal N, Nagala V, Gonzales-Marin C, Gillam DG, Bradshaw DJ, Burnett GR, Allaker RP. 2021. Interdental and subgingival microbiota may affect the tongue microbial ecology and oral malodour in health, gingivitis and periodontitis. J Periodontal Res 56:1174–1184. doi:10.1111/jre.12931 34486723

[27] Decat E, Cosyn J, De Bruyn H, Miremadi R, Saerens B, Van Mechelen E, Vermeulen S, Vaneechoutte M, Deschaght P. 2012. Optimization of quantitative polymerase chain reactions for detection and quantification of eight periodontal bacterial pathogens. BMC Res Notes 5:664. doi:10.1186/1756-0500-5-664 PMC3532386 23199017

[28] Horz HP, Vianna ME, Gomes B, Conrads G. 2005. Evaluation of universal probes and primer sets for assessing total bacterial load in clinical samples: general implications and practical use in endodontic antimicrobial therapy. J Clin Microbiol 43:5332–5337. doi:10.1128/JCM.43.10.5332-5337.2005 PMC1248440 16208011

[29] Gomes B, Berber VB, Kokaras AS, Chen T, Paster BJ. 2015. Microbiomes of endodontic-periodontal lesions before and after chemomechanical preparation. J Endod 41:1975–1984. doi:10.1016/j.joen.2015.08.022 PMC7061340 26521147

[30] Callahan BJ, McMurdie PJ, Rosen MJ, Han AW, Johnson AJA, Holmes SP. 2016. DADA2: high-resolution sample inference from Illumina amplicon data. Nat Methods 13:581–583. doi:10.1038/nmeth.3869 PMC4927377 27214047

[31] Douglas GM, Maffei VJ, Zaneveld JR, Yurgel SN, Brown JR, Taylor CM, Huttenhower C, Langille MGI. 2020. PICRUSt2 for prediction of metagenome functions. Nat Biotechnol 38:685–688. doi:10.1038/s41587-020-0548-6 PMC7365738 32483366

[32] Nakamura S, Shioya K, Hiraoka BY, Suzuki N, Hoshino T, Fujiwara T, Yoshinari N, Ansai T, Yoshida A. 2018. *Porphyromonas gingivalis* hydrogen sulfide enhances methyl mercaptan-induced pathogenicity in mouse abscess formation. Microbiology (Reading) 164:529–539. doi:10.1099/mic.0.000640 29488863

[33] Dewhirst FE, Chen T, Izard J, Paster BJ, Tanner ACR, Yu W-H, Lakshmanan A, Wade WG. 2010. The human oral microbiome. J Bacteriol 192:5002–5017. doi:10.1128/JB.00542-10 PMC2944498 20656903

[34] Takeshita T, Suzuki N, Nakano Y, Shimazaki Y, Yoneda M, Hirofuji T, Yamashita Y. 2010. Relationship between oral malodor and the global composition of indigenous bacterial populations in saliva. Appl Environ Microbiol 76:2806–2814. doi:10.1128/AEM.02304-09 PMC2863426 20228112

[35] Offenbacher S, Barros S, Mendoza L, Mauriello S, Preisser J, Moss K, de Jager M, Aspiras M. 2010. Changes in gingival crevicular fluid inflammatory mediator levels during the induction and resolution of experimental gingivitis in humans. J Clin Periodontol 37:324–333. doi:10.1111/j.1600-051X.2010.01543.x PMC2917904 20447255

[36] Hajishengallis G, Lamont RJ. 2021. Polymicrobial communities in periodontal disease: their quasi‐organismal nature and dialogue with the host. Periodontol 2000 86:210–230. doi:10.1111/prd.12371 PMC8957750 33690950

[37] Hasturk H, Kantarci A, Van Dyke TE. 2012. Oral inflammatory diseases and systemic inflammation: role of the macrophage. Front Immunol 3:118. doi:10.3389/fimmu.2012.00118 PMC3353263 22623923

[38] Schirmer M, Smeekens SP, Vlamakis H, Jaeger M, Oosting M, Franzosa EA, Ter Horst R, Jansen T, Jacobs L, Bonder MJ, Kurilshikov A, Fu J, Joosten LAB, Zhernakova A, Huttenhower C, Wijmenga C, Netea MG, Xavier RJ. 2016. Linking the human gut microbiome to inflammatory cytokine production capacity. Cell 167:1125–1136. doi:10.1016/j.cell.2016.10.020 PMC5131922 27814509

[39] Rai AK, Panda M, Das AK, Rahman T, Das R, Das K, Sarma A, Kataki AC, Chattopadhyay I. 2021. Dysbiosis of salivary microbiome and cytokines influence oral squamous cell carcinoma through inflammation. Arch Microbiol 203:137–152. doi:10.1007/s00203-020-02011-w 32783067

[40] Haffajee AD, Socransky SS, Patel MR, Song X. 2008. Microbial complexes in supragingival plaque. Oral Microbiol Immunol 23:196–205. doi:10.1111/j.1399-302X.2007.00411.x 18402605

[41] Pinacchio C, Scagnolari C, Iebba V, Santinelli L, Innocenti GP, Frasca F, Bitossi C, Scordio M, Oliveto G, Ceccarelli G, Antonelli G, Mastroianni CM, d’Ettorre G. 2020. High abundance of genus *Prevotella* is associated with dysregulation of IFN-I and T cell response in HIV-1-infected patients. AIDS 34:1467–1473. doi:10.1097/QAD.0000000000002574 32675560

[42] Mark Welch JL, Rossetti BJ, Rieken CW, Dewhirst FE, Borisy GG. 2016. Biogeography of a human oral microbiome at the micron scale. Proc Natl Acad Sci USA 113:E791–E800. doi:10.1073/pnas.1522149113 PMC4760785 26811460

[43] Aruni W, Chioma O, Fletcher HM. 2014. Filifactor alocis: the newly discovered kid on the block with special talents. J Dent Res 93:725–732. doi:10.1177/0022034514538283 PMC4126222 24898946

[44] Larsen JM. 2017. The immune response to *Prevotella* bacteria in chronic inflammatory disease. Immunology 151:363–374. doi:10.1111/imm.12760 PMC5506432 28542929

