# Supplementary material for: Sulfur metabolism and immune-microbial networks across oral niches in periodontal disease
Source: Microbiol Spectr. 2026 Aug 31;14(10):e03008-25. doi: 10.1128/spectrum.03008-25 (PMC13688991; doi:10.1128/spectrum.03008-25)
Supplement: Supplementary file 1 [file spectrum.03008-25-s0003.docx]

**Supplementary Methods**

**S1. Study design, participants, and ethics**

Participants (18–70 y; ≥20 teeth) were recruited between February 2013–May 2015 at Queen Mary University of London and the Royal London Dental Hospital (Barts Health NHS Trust). Allocation to low plaque health (LPH), high plaque health (HPH), gingivitis, or chronic periodontitis followed clinical criteria detailed in (1). Ethical approval: London Central REC 12/LO/1611. Written informed consent was obtained; the study adhered to the Declaration of Helsinki and STROBE.

Pre-visit restrictions: Participants abstained from eating and oral hygiene for ≥2 h, and from malodorous foods (e.g., garlic, onion, coffee) for ≥12 h prior to sampling.

**S2. Sampling and processing**

**S2.1 Sample collection sequence**

Unless clinical constraints dictated otherwise, the sequence was: (i) oral-headspace sampling, (ii) unstimulated saliva, (iii) tongue dorsum biofilm, (iv) subgingival plaque (deepest site per quadrant; buccal + lingual), (v) interdental supragingival plaque (mesial + distal). Biofilm/plaque specimens were placed into TE buffer (Tris–EDTA, pH 7.2; Sigma-Aldrich) on ice and stored at −80 °C.

**S2.2 Oral-headspace VSC sampling (OralChroma)**

Participants sat upright and breathed through their nose with their mouth closed for approximately one minute. A sterile FEP mouthpiece (Cole-Parmer, UK) with a 20ml PTFE syringe (NORM-JECT®, Henke-Sass Wolf, Germany) attached was then inserted into the mouth till the mouthpiece reached the back half of the tongue. Ensuring that the participants held the mouthpiece with the lips closed (but not sealed) around the mouthpiece, they were instructed to take a deep inhalation through their nose and hold for 5 seconds, during which approximately 15ml of mouth air sample was withdrawn. A 1 mL aliquot of this sample was injected into the Oral Chroma for analysis.

**S3. Functional amino-acid degradation assays (headspace GC-FPD)**

**S3.1 Protein quantification**

Total protein was measured by bicinchoninic acid (BCA) assay (Pierce BCA, ThermoFisher Scientific) in microplate format using supplied BSA standards.

**S3.2 Incubation conditions**

Aliquots (25 µL) of saliva, tongue, subgingival, supragingival, and interdental samples were incubated at 37 °C with gentle shaking (~80 rpm) in sealed 10 mL glass headspace vials containing 0.5 mL of 0.5% (w/v) L-cysteine (1 h) or L-methionine (24 h) (Sigma, UK). Metabolism was arrested by injecting 1 mL absolute ethanol immediately post-incubation. All amino acid degradation assays were performed under anaerobic conditions. Vials were prepared and sealed within an anaerobic chamber prior to incubation. Incubation times were empirically optimized to obtain robust, non-saturating GC-FPD signals within the linear detection range. Cysteine-derived H_2_S production reached detectable and near-plateau levels within 1 hour, whereas methionine-derived CH_3_SH production required extended incubation (24 hours) to generate reproducible, quantifiable signals. These differences likely reflect known enzymatic kinetics, as cysteine desulfhydrase activity typically proceeds more rapidly than methionine γ-lyase (MGL)-mediated methanethiol generation. Although incubation times were optimized based on preliminary signal testing, full time-course kinetic analyses were not performed, and therefore these measurements represent endpoint functional readouts rather than quantitative enzyme-rate comparisons.

**S3.3 Gas chromatography (instrument and run conditions)**

Headspace VSCs were analysed on an Agilent 6890N GC (Agilent Technologies, Edinburgh, UK) equipped with a sulfur-specific flame photometric detector (FPD) and Chromosil 330 packed column operated isothermally at 60 °C with helium carrier at 45 mL min^-1^. FPD settings: 175 °C; H_2_ 50 mL min^-1^, air 75 mL min^-1^, N_2_ makeup 15 mL min^-1^. Injections used a gas-sampling valve with sulfinert-treated loops: 1 mL for CH_3_SH and 250 µL for H_2_S.

**S3.4 Calibration**

Prior to runs, the GC was calibrated with a Kin-Tek 491M gas standards generator (Kin-Tek Laboratories, La Marque, TX) fitted with H_2_S and CH_3_SH permeation tubes. Calibration curves were constructed, and limits of detection were derived from fitted log–log regressions. Assay blanks (substrate-free), loop blanks, and standard re-checks were included per batch.

**S4. Subgingival VSC quantification (headspace GC-FPD)**

**S4.1 Sample preparation and headspace sampling**

GCF-wetted paper strips (Periocol/Pericol) from subgingival sites were sealed in headspace vials, incubated at 80 °C for 2 min, and 2 mL of headspace was drawn with a gas-tight syringe (NORM-JECT®) and injected into the GC-FPD under the same chromatographic conditions as S3.

**S4.2 Simulated-strip standard curves**

To reproduce the sampling workflow, filter strips were dipped 5 mm below the meniscus of 2 mL standard dilutions prepared from sodium sulfide nonahydrate (290 mM) or sodium thiomethoxide (230 mM) stock solutions (Sigma) for 10 s, then sealed in glass crimp-top vials with 300 µL inserts (Supelco/Sigma). Immediately prior to sampling, vials were acidified with 40 µL 1 M phosphoric acid to release VSCs.

To characterise strip-dependent adsorption and analyte loss, parallel neat-solution controls were prepared in which equivalent standard solutions were acidified and analysed in the absence of absorbent material. Comparison of recoverable headspace VSCs in the presence and absence of strips enabled assessment of strip-related attenuation and informed interpretation of clinical measurements.

Peak areas were interpolated on log–log calibration curves generated from known standards; linear regression provided interpolation functions and LOD estimates. All samples and standards were processed in parallel. Replicate measurements across a range of concentrations were used to estimate within-run variability. All standards and clinical samples were processed in parallel and analysed within a single GC–FPD run. Blank control vials processed in parallel showed no detectable signal. Peak areas from clinical samples were interpolated using the same simulated-strip calibration curves generated under identical analytical conditions, such that both standards and samples were subject to the same strip-dependent adsorption and release effects.

| **Parameter** | **Filter paper strips (Whatman / Periocol)** | **Basis / definition** |
| --- | --- | --- |
| **Lower limit of detection (LLOD)** | ~0.3 ng/mg (≈ 7 µM) | Lowest analyte load consistently distinguishable from blanks with reproducible non-zero signal |
| **Recovery relative to neat solution** | ~30–60% (concentration-dependent) | Estimated from paired neat vs neat + strip experiments, reflecting strip adsorption and non–acid-labile fraction |
| **Intra-run CV (above LLOD)** | ~20–60% | Calculated from replicate measurements (n=6) within a single GC–FPD run |
| **Intra-run CV (near LLOD)** | >100% | Expected behaviour for volatile analytes close to detection limit |
| **Blank signal** | Not detected | Control vials processed in parallel showed zero or baseline signal |
| **Linearity (qualitative)** | Monotonic across ~2 orders of magnitude | Based on increasing sqrt peak area with analyte load; formal R² not reported for strip-attenuated standards |

**Supplementary Table S1. Analytical performance characteristics of filter strip–based volatile sulfur compound (VSC) measurements.**

**S5. Cytokine multiplex immunoassay (GCF)**

**S5.1 Collection and elution**

GCF for cytokines was collected using sterile Whatman 3MM or Periocol strips (two per tooth, buccal and lingual). Strips were pooled and eluted by adding 150 µL TE to the storage tube, wetting all strips, holding 1 min on ice, then centrifuging at 3,500 g, 4 °C, 5 min. Eluates were transferred to microcentrifuge tubes and stored at −80 °C.

**S5.2 Assay and acquisition**

A 20-analyte fluorimetric bead immunoassay (FlowCytomix Human Inflammation 20-plex, eBioscience) was run per manufacturer instructions and acquired on a BD FACSCanto II (BD Biosciences). Briefly, a standard series was prepared from lyophilized standards; 25 µL bead mix was added to samples/standards/blanks, followed by 50 µL Biotin-Conjugate, incubated 2 h RT protected from light. Washes: 1 mL assay buffer, 200 *g*, 5 min, supernatant reduced to ~100 µL, repeated. 50 µL diluted Streptavidin-PE was added (1 h RT), washed twice, resuspended in 500 µL assay buffer, and acquired.

Standard curves used five-parameter logistic (5-PL) fits; concentrations were computed in FlowCytomix Pro 3.0. Plate blanks, duplicate samples, and internal controls were included on each run. Samples outside standard ranges were re-assayed when volume allowed.

**S6. DNA extraction**

**S6.1 Sample disruption and lysis**

Tongue and plaque samples were dislodged from swabs/brushes by adding 3–5 sterile glass beads and vortexing 20 s. Saliva (1 mL) was pelleted at >10,000 *g*, 10 min, 4 °C and resuspended in TE. DNA was extracted using the MasterPure Gram-Positive DNA kit (Epicentre, Madison, USA) with an initial overnight 37 °C incubation including 1 µL Ready-Lyse lysozyme per sample.

**S7. Quantitative PCR (qPCR)**

**S7.1 Targets, standards, and culture conditions**

Absolute abundances of *Fusobacterium nucleatum, Tannerella forsythia, Aggregatibacter actinomycetemcomitans*, and *Porphyromonas gingivalis* were quantified by SYBR Green qPCR using published 16S or rpoB primers. Genomic DNA from pure cultures served for standard curves (Ct to CFU equivalents). Strains were grown in an anaerobic chamber (MAC MG500; Don Whitley Scientific, UK; 80% N_2_/10% H_2_/10% CO_2_, 37 °C). Growth media was Blood agar base No.2 with 5% v/v defibrinated horse blood for all strains, with the supplement of 0.001% w/v N-acetylmuramic acid for *T. forsythia.* Primer sequences are listed in table below.

| *Organism* | Primers | References |
| --- | --- | --- |
| *Aggregatibacter actinomycetemcomitans* | F: CTTACCTACTCTTGACATCCGAA  R: ATGCAGCACCTGTCTCAAAGC | (2) |
| *Fusobacterium* spp. | F: AAGCGCGTCTAGGTGGTTATGT  R: TGTAGTTCCGCTTACCTCTCCAG | (3) |
| *Tannerella forsythia* | F: GGGTGAGTAACGCGTATGTAACCT  R: ACCCATCCGCAACCAATAAA | (4) |
| *Porphyromonas gingivalis* | F: GGAAGAGAAGACCGTAGCACAAGGA  R: GAGTAGGCGAAACGTCCATCAGGTC | (5) |

**Supplementary Table S2. Primer sequences used for the quantitative PCR based detection of periodontal pathogens.**

**S7.2 Reaction setup and cycling**

Reactions were assembled in 96-well plates (Roche) at 20 µL: 2× LightCycler® FastStart DNA Mastermix (Roche), 1 µM of each primer, 2 µL template, water to volume. Clinical DNA was diluted 1:10 to mitigate inhibition. Plates were sealed, centrifuged (1,500 g, 10 s), and run on LightCycler LC480 with polymerase activation (95 °C, 5 min), then 40 cycles: 95 °C, 10 s; Ta, 5 s; 72 °C extension (~1 s/25 bp). SYBR fluorescence was recorded at 76 °C, 1 s.

Melt curve: 95 °C, 30 s → 65 °C, 30 s → acquisition at 99 °C; ramp 4.4 °C s^-1^, final step 0.1 °C s^-1^. Data were processed in LightCycler v1.5. The Absolute Fit Points method was used; 35 cycles served as last-cycle cutoff. Baselines were adjusted manually to optimize Ct sensitivity in positives. No-template and negative sample controls were included; all reactions were duplicate. For the universal 16S standard, genomic DNA from *P. gingivalis* cultures was used. This organism has a well-characterised genome containing four copies of the 16S rRNA gene, allowing conversion of measured 16S gene copy numbers to estimated bacterial cell equivalents. Species-specific CFU equivalents were expressed relative to universal 16S where indicated. Proportions were used for normalization only, given dependence on 16S copy-number variation and universal-primer breadth (Horz et al., 2005).

**Supplementary Figure S1. UpSet plot illustrating overlap between clinical/VSC (n = 92), sequencing (n = 54), and cytokine (n = 45) datasets. Functional data were not included in the network analysis.**

| **Predictor** | **GCF H₂S β (SE)** | **p** | **Oral H₂S β (SE)** | **p** | **Oral CH₃SH β (SE)** | **p** |
| --- | --- | --- | --- | --- | --- | --- |
| Intercept | 1.675 (0.466) | 0.0007*** | 3.017 (0.652) | <0.001*** | 0.897 (0.540) | 0.101 |
| Diagnosis: HPH | 0.579 (0.576) | 0.319 | 0.629 (0.823) | 0.447 | 0.179 (0.683) | 0.794 |
| Diagnosis: Gingivitis | 0.618 (0.496) | 0.217 | 0.267 (0.759) | 0.726 | 0.594 (0.630) | 0.348 |
| **Diagnosis: Periodontitis** | **1.235 (0.554)** | **0.030*** | 0.642 (0.847) | 0.451 | 1.147 (0.703) | 0.107 |
| Age (z) | 0.096 (0.192) | 0.620 | 0.180 (0.290) | 0.537 | -0.043 (0.240) | 0.859 |
| Sex (Male) | 0.120 (0.314) | 0.705 | 0.521 (0.479) | 0.280 | 0.698 (0.398) | 0.083 |
| Plaque index (z) | -0.110 (0.178) | 0.539 | 0.263 (0.285) | 0.358 | 0.256 (0.236) | 0.282 |
| **Subgingival load (z)** | 0.761 (0.449) | 0.095 | 0.187 (0.239) | 0.437 | **0.400 (0.198)** | **0.047*** |

**Supplementary Table S3. Multivariable linear models for volatile sulfur compounds**

| **Cytokine** | **β (Periodontitis)** | **SE** | **t** | **p-value** | **FDR-adjusted p** |
| --- | --- | --- | --- | --- | --- |
| **IL6** | 2.35 | 0.80 | 2.95 | 0.0057 | 0.342 |
| **IL17a** | 2.01 | 0.88 | 2.28 | 0.029 | 0.582 |
| IL13 | 0.66 | 0.34 | 1.98 | 0.056 | 0.732 |
| GCSF | 1.15 | 0.60 | 1.92 | 0.063 | 0.732 |
| MCP1 | 0.82 | 0.45 | 1.83 | 0.076 | 0.732 |
| IL8 | 1.59 | 0.96 | 1.66 | 0.105 | 0.732 |
| TNFa | -1.75 | 1.14 | -1.54 | 0.133 | 0.732 |
| IL12p70 | -1.41 | 0.90 | -1.56 | 0.127 | 0.732 |
| IFNg | -1.22 | 0.84 | -1.44 | 0.158 | 0.732 |
| IL1a | -2.12 | 1.97 | -1.07 | 0.290 | 0.807 |
| IL10 | 0.90 | 0.85 | 1.06 | 0.295 | 0.807 |
| MIP1b | 1.26 | 0.96 | 1.32 | 0.195 | 0.732 |
| MIP1a | -0.04 | 0.89 | -0.04 | 0.967 | 0.981 |
| IL4 | -0.63 | 0.65 | -0.97 | 0.341 | 0.807 |
| ICAM1 | 0.05 | 0.56 | 0.09 | 0.929 | 0.966 |
| IFNa | 1.00 | 1.16 | 0.87 | 0.392 | 0.807 |
| CXCL10 | -0.13 | 0.28 | -0.47 | 0.639 | 0.966 |
| LAP | 0.73 | 0.48 | 1.53 | 0.135 | 0.732 |
| Eselectin | 0.11 | 0.69 | 0.16 | 0.877 | 0.966 |
| IL1b | 0.14 | 1.06 | 0.13 | 0.899 | 0.966 |

**Supplementary Table S4. Multivariable linear models for GCF cytokines**

**Supplementary Figure S2.** **Predicted abundance of microbial carbon–sulfur lyases across oral niches and disease states.**

**References**

1.	Stephen AS, Dhadwal N, Nagala V, Marin CG-, Gillam DG, Bradshaw DJ, Burnett GR, Allaker RP. 2021. Interdental and subgingival microbiota may affect the tongue microbial ecology and oral malodour in health , gingivitis and periodontitis. Journal of periodontal research 1–11.

2.	Maeda H, Fujimoto C, Haruki Y, Maeda T, Kokeguchi S, Petelin M, Arai H, Tanimoto I, Nishimura F, Takashiba S. 2003. Quantitative real-time PCR using TaqMan and SYBR Green for Actinobacillus actinomycetemcomitans , Porphyromonas gingivalis , Prevotella intermedia , tetQ gene and total bacteria. FEMS Immunology & Medical Microbiology 39:81–86.

3.	Martin FE, Nadkarni M a., Jacques N a., Hunter N. 2002. Quantitative Microbiological Study of Human Carious Dentine by Culture and Real-Time PCR: Association of Anaerobes with Histopathological Changes in Chronic Pulpitis. Journal of clinical microbiology 40:1698–1704.

4.	Shelburne CE, Prabhu A, Gleason RM, Mullally BH, Coulter WA. 2000. Quantitation of Bacteroides forsythus in subgingival plaque: Comparison of immunoassay and quantitative polymerase chain reaction. Journal of Microbiological Methods 39:97–107.

5.	Park S-N, Park J-Y, Kook J-K. 2011. Development of Porphyromonas gingivalis-specific quantitative real-time PCR primers based on the nucleotide sequence of rpoB. Journal of microbiology (Seoul, Korea) 49:315–9.
